# Residual eDNA in eRNA Extracts Skews eRNA‐Based Biodiversity Assessment: Call for Optimised DNase Treatment

**DOI:** 10.1111/1755-0998.70102

**Published:** 2026-01-19

**Authors:** Fuwen Wang, Wei Xiong, Xuena Huang, Shiguo Li, Aibin Zhan

**Affiliations:** ^1^ Research Center for Eco‐Environmental Sciences Chinese Academy of Sciences Beijing China; ^2^ University of Chinese Academy of Sciences, Chinese Academy of Sciences Beijing China

**Keywords:** biodiversity assessment, DNase treatment, environmental DNA, environmental RNA, false positives

## Abstract

Environmental RNA (eRNA) metabarcoding has rapidly emerged as a powerful tool for assessing contemporary biodiversity patterns across diverse ecosystems. However, the potential for false positive detections caused by co‐extracted environmental DNA (eDNA) remains unquantified. Distinguishing true signals from false positives caused by residual eDNA is a technical challenge in eRNA‐based metabarcoding. To address this issue, we employed a freshwater river receiving treated effluent from a wastewater treatment plant as a model system. In such settings, eDNA in the treated effluent can lead to the detection of non‐local species (e.g., marine taxa). Treated effluent typically contains minimal or no eRNA, making it well‐suited for evaluating the influence of eDNA carryover. By comparing DNase‐treated and untreated eRNA samples, we assessed the impact of residual eDNA on fish species richness and community composition. Our results showed that omitting DNase treatment significantly inflated taxonomic richness, with untreated samples detecting a conservative estimate of over 25% more taxa per site. Fold‐change analysis revealed that residual eDNA inflated taxon abundances in both high‐ and low‐abundance taxa, with some showing over 10‐fold increases. Community composition analyses revealed clear clustering between treated and untreated samples, highlighting substantial shifts driven by residual eDNA. These findings demonstrate that co‐extracted eDNA can severely distort eRNA‐based biodiversity estimates, leading to false positives and misrepresented contemporary community profiles. We recommend further evaluation of DNase treatment parameters, including enzyme concentration, incubation time and treatment times, and the adoption of optimised protocols to standardise and improve the accuracy of eRNA‐based biodiversity monitoring.

## 1 Introduction

The use of environmental nucleic acids (eNA), including environmental DNA (eDNA) and RNA (eRNA), has rapidly advanced as a transformative tool across ecological and environmental disciplines (Thomsen and Willerslev 2015; Pawlowski et al. 2021; Sun et al. 2025). eNA, which captures genetic material shed by organisms into their surroundings, enables highly sensitive, non‐invasive and scalable biodiversity assessments across diverse ecosystems (Littlefair et al. 2022; Xia et al. 2024; Zhang et al. 2024). Because DNA can persist in the environment for weeks or even much longer under certain conditions, such as millions of years in sediment, eDNA reflects both current and historical biological presence (Sakata et al. 2020; Angeles et al. 2023). In contrast, due to its relatively rapid degradation, eRNA provides a more precise snapshot of metabolically active organisms (Veilleux et al. 2021; Wang, Xiong, Liu, et al. 2025). Emerging research demonstrates that eRNA metabarcoding improves the temporal resolution of biodiversity assessments by distinguishing viable organisms from residual or legacy genetic signals (Littlefair et al. 2022; Wang, Xiong, Liu, et al. 2025). Thus, eDNA and eRNA offer complementary insights: eDNA captures the historical and recent presence of organisms, while eRNA reveals real‐time biological activity. The combined use of both methods significantly enhances ecosystem monitoring, invasive species detection and the assessment of ecological health.

Given the widespread adoption and exceptional sensitivity of both eDNA and eRNA methodologies, it is imperative to rigorously identify, characterise and address potential sources of error to ensure the reliability and validity of their applications across diverse scientific disciplines (Darling and Mahon 2011; Ficetola et al. 2015; Wang et al. 2025a; Zhan 2025). Due to the earlier development and broader adoption of eDNA‐based approaches, the scientific community has gained considerable insights into common pitfalls such as false positives and false negatives (Beng and Corlett 2020; Burian et al. 2021; Diana et al. 2021). Many of these technical challenges have been mitigated through iterative improvements in sampling techniques (Xiong and Zhan 2018; Dickie et al. 2018), experimental and analytical protocols (Strand et al. 2011; Zhan, Xiong, et al. 2014; Sun et al. 2015; Dairawan and Shetty 2020), contamination controls (Bohmann and Lynggaard 2023) and bioinformatic analysis (Xiong and Zhan 2018; Mathon et al. 2021). In contrast, errors associated with eRNA remain less well characterised. From a methodological perspective, once eRNA is reverse transcribed into complementary DNA (cDNA), the sources of error during downstream processes, such as amplification, sequencing and data analysis, closely resemble those encountered in eDNA workflows. Therefore, the principal novel sources of error and potential technical solutions in eRNA studies should be prioritised in the initial experimental steps preceding and including reverse transcription (Wang et al. 2025a; Zhan 2025).

To date, error identification and potential solutions primarily focus on the fate of eRNA in natural environments (Scriver et al. 2023 and references therein), methods for its extraction and storage (Jo 2023), and protocol optimizations and technical improvements for reverse transcription (Wang et al. 2025a). Environmental factors such as temperature fluctuations, ultraviolet radiation and ribonuclease (RNase) activity contribute to the rapid degradation of eRNA, often leading to false negatives by rendering target sequences undetectable even when source organisms are present (Scriver et al. 2023). To improve recovery efficiency of trace amounts of eRNA from environmental samples, increasing the lysis buffer volume and using more permeable filters (e.g., GF/A) have proven effective (Jo 2023). Moreover, the use of RNAlater can preserve eRNA on filters for at least six days at 4°C–20°C, providing a practical alternative to freezing in field conditions (Jo 2023). Similarly, the BioDry method enables ambient‐temperature storage of microbial RNA for up to 30 days with minimal degradation (Tuorto et al. 2015). After eRNA extraction, reverse transcription strategies also critically influence the accuracy of eRNA‐based analyses, particularly in minimising false negatives (Wang et al. 2025a). Random hexamer primers, which anneal at multiple sites along RNA templates, enhance sensitivity by recovering rare or fragmented transcripts more effectively than oligo (dT) or taxa‐specific primers, which target more limited regions (Wang et al. 2025a). Combining random hexamers with oligo (dT) primers can broaden taxonomic coverage and reduce amplification bias. For non‐polyadenylated RNA species, random priming remains the preferred method (Wang et al. 2025a). Altogether, a comprehensive understanding and careful control of these upstream factors are essential to maximise the functional and temporal resolution of eRNA‐based studies, while minimising artefacts that could compromise ecological or biomedical interpretations.

One of the additional error sources in eRNA‐based studies is the unintended co‐extraction of eDNA in final RNA preparations. Technically, even when using RNA‐specific reagents, such as those included in commercial kits designed for RNA isolation (e.g., TRIzol or silica column‐based methods), DNA contamination can still occur (Chai et al. 2005; Nguyen et al. 2024). This is because many RNA extraction protocols rely on chaotropic agents or binding conditions that do not completely distinguish between RNA and DNA, particularly when the DNA is fragmented or exists in extracellular forms, as is common in environmental samples (Lebuhn et al. 2016; Ali et al. 2017). Furthermore, RNA‐specific reagents may not fully eliminate DNA carryover, with residual DNA often retained on silica membranes or remaining in the aqueous phase during phase separation (Ali et al. 2017; Nguyen et al. 2024). The presence of DNA in RNA extracts has been widely reported, especially in gene expression studies. For instance, genomic DNA contamination has been shown to yield false‐positive signals in RT‐qPCR assays targeting long non‐coding RNAs such as *MALAT1*, where even trace levels of DNA were sufficient to misrepresent transcript abundance (Markou et al. 2021). Similarly, in RNA‐seq analyses, spiking experiments revealed that as little as 1%–2% DNA contamination can artificially inflate low‐abundance transcript levels, skew differential expression outcomes and bias downstream functional enrichment analyses (Li et al. 2022). These examples collectively underscore the substantial impact that residual DNA in RNA extracts can have on results and their subsequent interpretation. Owing to the common occurrence of DNA residual in RNA extracts, it is reasonable to hypothesise that such residual could also cause skewed results in eRNA‐based biodiversity assessment. Nonetheless, many eRNA studies to date do not clearly report whether DNase treatment has been applied to eliminate co‐extracted DNA. Thus, it raises significant concerns regarding the potential inclusion of eDNA in eRNA datasets, which can lead to false positive detections and inflated estimates of species diversity. Without rigorous DNase digestion and subsequent validation of DNA removal, confidently attributing detected sequences exclusively to RNA remains problematic, thereby compromising the accuracy and reliability of biodiversity inferences derived from eRNA analyses. Addressing this critical methodological gap is essential to standardise eRNA workflows and enhance the robustness of nucleic acid‐based ecological and environmental monitoring.

Despite the growing application of eRNA metabarcoding to achieve contemporary biodiversity assessments, the extent to which residual eDNA contamination influences eRNA‐based detection remains poorly understood. This study aims to evaluate the impact of co‐extracted eDNA on biodiversity estimates derived from eRNA metabarcoding under ecologically realistic conditions. Using fish communities in a freshwater ecosystem as a case study, we aim to compare DNase‐treated and untreated eRNA samples to quantify the degree of false positives introduced by residual eDNA. Specifically, we investigate how residual eDNA affects species richness, community composition and detection across abundance levels. The results obtained in this study are expected to provide references to improve the accuracy and reliability of eRNA‐based ecological monitoring.

## 2 Materials and Methods

### 2.1 Sampling Design

**FIGURE 1:**
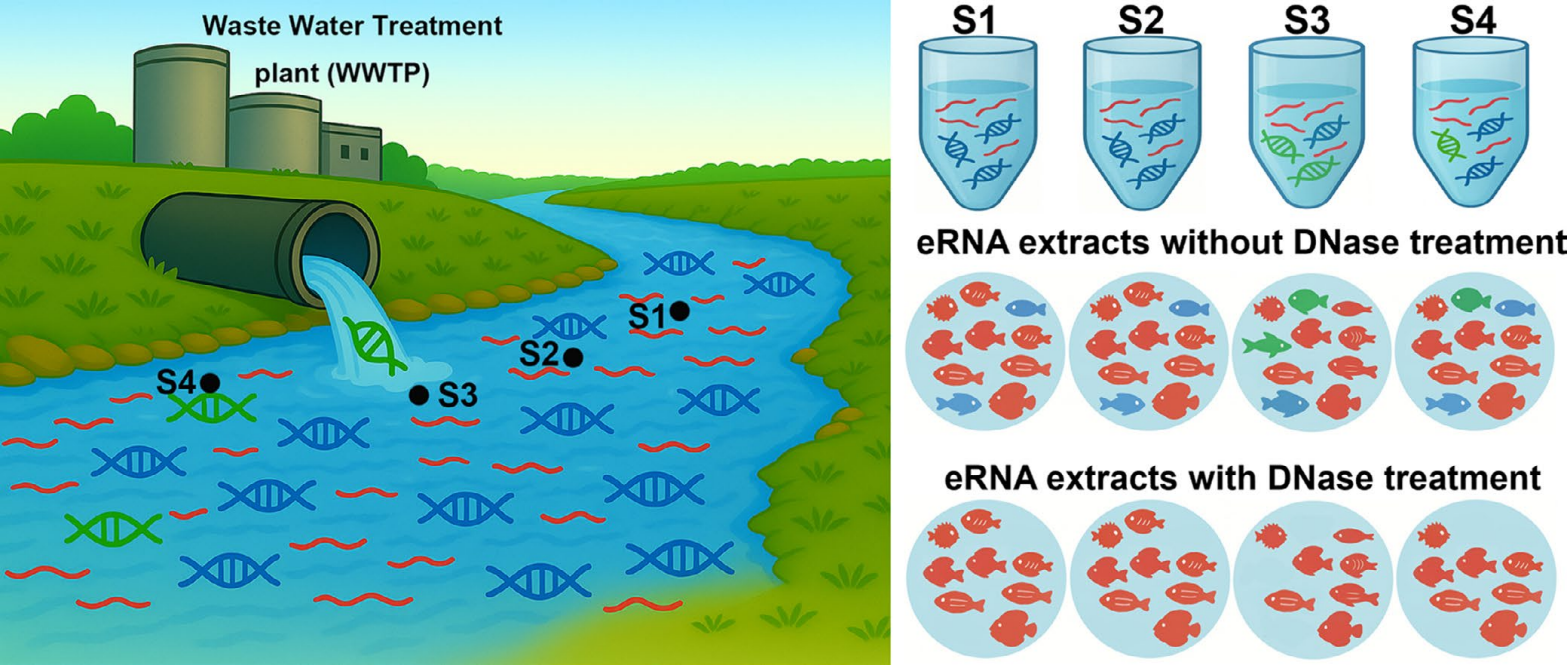
Schematic illustration of the experimental design and eRNA analysis workflow. The freshwater river receives treated effluent from a wastewater treatment plant (WWTP), which contains environmental DNA (eDNA) from human activities (highlighted in green) but little to no environmental RNA (eRNA). Sampling Sites S1 and S2, located upstream of the WWTP, are minimally affected by WWTP‐derived eDNA, whereas Sites S3 and S4, situated at the effluent discharge point and downstream, are more strongly influenced by WWTP‐derived eDNA pollution. By identifying non‐local fish taxa introduced via eDNA pollution from human activities, we obtained a conservative estimate of the extent to which residual eDNA in eRNA extracts affects eRNA‐based biodiversity assessments.

A key technical challenge in assessing the influence of residual eDNA is accurately distinguishing species detections resulting from co‐extracted eDNA contamination from those representing true biological signals captured through eRNA‐based metabarcoding. To address this, we employed a freshwater river receiving treated effluent from a wastewater treatment plant (WWTP) as a model system (39.88905° N, 116.55418° E; Figure 1). In such a system, the discharge point and the downstream area are contaminated with eDNA derived from the WWTP, resulting in the detection of species not naturally present in the local aquatic ecosystem (e.g., marine species) (Xiong et al. 2024; Zhan 2025). However, owing to rapid RNA degradation during the treatment process, eRNA in the treated effluent is below detectable levels (Xiong et al. 2024). As a result, we expect that if residual eDNA in eRNA extracts remains and further contributes to false positives, we would observe significantly higher numbers of taxa, particularly marine taxa (e.g., commercially consumed species), originating from eDNA contamination derived from the WWTP (Xiong et al. 2024). This design conservatively estimates residual eDNA influence by detecting effluent‐derived taxa; we avoided mock communities as they inadequately replicate the complexity and in situ degradation states of natural environmental matrices. Furthermore, mock communities do not capture the real‐world co‐occurrence of both high‐abundance and low‐abundance taxa. By using a field‐based system with known eDNA contamination and expected eRNA absence, our approach allows us to test contamination effects under ecologically realistic conditions, thereby improving the relevance and applicability of our findings to natural monitoring scenarios.

According to the experimental design, water samples were collected from four sites along the Tonghui River, which serves as the receiving water body for the largest WWTP in Beijing. Our previous study demonstrated that, although advanced wastewater treatment technologies have been employed to eliminate a wide range of contaminants in this WWTP, diverse fish taxa were still detected in eDNA collected from the fully treated effluent, whereas no fish taxa were recovered from eRNA (Xiong et al. 2024). Sampling locations were established both upstream and downstream of the effluent discharge point (Figure 1). Specifically, two sites (S1 and S2) were positioned upstream of the discharge point, one site (S3) at the discharge point and one site (S4) downstream. At each sampling site, six independent 1 L water samples were collected in sterile bottles and subsequently pooled to form a single 6 L composite sample. This strategy ensures an adequate quantity of eRNA for downstream analyses while reducing the influence of random sampling variability. A total of six composite replicates were collected at each site, and the collection and filtration of each composite replicate were completed within 20 min (Wang et al. 2025b). In addition, at each site, we collected 1 L of sterile distilled water using the same sampling procedure to serve as a blank control. The smaller volume (1 L) of blank controls, which is commonly used in eDNA/eRNA studies (e.g., van der Loos and Nijland 2021), was specifically selected to increase the sensitivity for detecting accidental contamination introduced during sampling and filtration, as larger volumes could dilute low‐level contaminants below detection limits.

All water samples, including the blank controls, were immediately filtered on‐site through mixed cellulose ester (MCE) membranes with a pore size of 0.45 μm (Millipore, USA). The filtered samples were transferred into 2 mL sterile, enzyme‐free cryovials (BioShark, China) and immediately stored in liquid nitrogen. Subsequent nucleic acid extraction was completed within one week.

### 2.2 Nucleic Acid Extraction, Reverse Transcription, PCR Amplification and Sequencing

Total eRNA was extracted individually from all filtered membranes using the TRIzol Kit (Thermo Fisher Scientific, USA), yielding 28 samples in total (4 sampling sites × 6 replicates + 4 sampling sites × 1 blank control). Each extract was evenly divided into two aliquots: one fully treated with the TURBO DNA‐free Kit (Thermo Fisher Scientific, USA) to remove residual eDNA and the other left untreated. The DNase‐treated eRNA was then tested by qPCR to confirm that no residual DNA was detectable. To assess RNA quality and quantity, all samples were analysed using a NanoDrop UV spectrophotometer (Thermo Scientific Inc., USA) and 1.5% agarose gel electrophoresis. To ensure complete eDNA removal, the extracts were treated with 1 U of DNase for every 100 ng total nucleic acid and incubated at 37°C for 30 min. All subsequent steps were carried out following the manufacturer's protocol for the TURBO DNA‐free Kit.

Following the recommendations of Wang et al. (2025a), reverse transcription was performed identically on both treated and untreated sample groups using random pentamer (5‐mer) primers and the PrimeScript II First Strand cDNA Synthesis Kit (TaKaRa Bio Inc., Shiga, Japan). Compared with commonly used random hexamer primers, the use of random pentamer primers can effectively improve the stability and diversity of community detection (Zhan Lab, unpublished data). For each reaction, a primer mix was first prepared containing 1 μL of 50 μM primer solution, 1 μL of 10 mM dNTP mixture and 300 ng of extracted eRNA in a total volume of 10 μL. This mixture was incubated at 65°C for 5 min and then quickly cooled on ice. Next, 4 μL of 5× PrimeScript II Buffer, 0.5 μL of RNase inhibitor (40 U/μL), 1 μL of PrimeScript II RTase (200 U/μL), and 4.5 μL of RNase‐free water were added, bringing the final reaction volume to 20 μL. Reverse transcription was carried out under the following thermal cycling conditions: 30°C for 10 min, 42°C for 45 min, followed by enzyme inactivation at 95°C for 5 min.

Subsequently, products of reverse transcription for both groups were identically amplified using fish‐specific Metafish primers targeting mitochondrial 12S rRNA regions using the protocol recommended by Xiong et al. (2024). Briefly, PCRs were prepared in a total volume of 25 μL, consisting of 2.5 μL of 10× PCR Buffer (Takara, Japan), 2 μL of dNTPs (2.5 mM each), 1.5 μL each of forward and reverse primers (10 μM), 2 U of Taq polymerase (Ex Taq, Takara), 1 μL of cDNA template and 16.5 μL of ddH_2_O. To reduce random sampling errors, each technical replicate included eight parallel PCR mixes (Zhan et al. 2013; Zhan, He, et al. 2014). The PCR amplification was performed under the following thermal cycling conditions: initial denaturation at 95°C for 5 min; followed by 35 cycles of 95°C for 30 s, annealing at 62.5°C for 30 s and extension at 72°C for 30 s; with a final extension at 72°C for 10 min. After amplification, the products from the eight parallel reactions within each technical replicate were pooled. All pooled PCR products were examined by agarose gel electrophoresis, purified using the Sanprep Column PCR Product Purification Kit (Sangon Biotech, China), and then sequenced on the Illumina NovaSeq 6000 platform using paired‐end reads (2 × 150 bp). All blank controls underwent the same procedures as the samples; although no amplification was detected on agarose gels after PCR, these blanks were also subjected to sequencing.

### 2.3 Bioinformatics Analysis

Bioinformatic analysis followed the methods described by Xiong and Zhan (2018), with several minor modifications. Briefly, raw sequence data were processed using a Galaxy‐based pipeline (https://dmap.denglab.org.cn/) (Feng et al. 2017). The ‘detect barcodes (FASTQ)’ tool was first used to demultiplex the FASTQ files (maximum number of errors in barcode = 1.5). Primers were then removed using the ‘trim primer’ function, allowing up to 1.5 mismatches and a maximum starting position of 1. Trimmed sequences were merged with the ‘flash’ tool, requiring a minimum overlap of 10 bp. Then sequences were filtered by removing ones with Phred scores (Q) lower than 35 or containing any undetermined nucleotide ‘N’ using the ‘btrim’ tool. The filtered reads were clustered into zero‐radius operational taxonomic units (ZOTUs) using the ‘Unoise for FASTA to generate ZOTUs’ function, with a minimum abundance threshold of 8. This process generated both a species abundance table and representative sequences for each ZOTU (Edgar 2016).

To assign taxonomy, ZOTU sequences were aligned against the MitoFish database (version 4.09, https://mitofish.aori.u‐tokyo.ac.jp/) using Seed version 2.1.2 (Větrovský et al. 2018; Zhu et al. 2023). The assigned taxonomic identities were then manually curated following the methods of Zhang et al. (2022) and Wang et al. (2025a). In general, for each ZOTU, alignment hits with the lowest (and identical) e‐values were manually examined. If all top hits corresponded to the same species, the ZOTU was assigned at the species level. If the top hits represented different species within the same genus, the ZOTU was assigned to that genus (i.e., Genus sp.). This hierarchical approach was applied analogously at higher taxonomic levels when necessary. All blank controls were processed using identical bioinformatic procedures, and no valid fish species were detected in any of the four blank controls, demonstrating that the entire workflow, including field sampling, eRNA extraction, DNase treatment, PCR and sequencing, was contamination‐free.

### 2.4 Influence of Residual eDNA on Biodiversity Parameters

To evaluate the impacts of co‐extracted eDNA residues in eRNA extracts on biodiversity, we compared the number of taxa detected between the DNase‐treated and untreated groups across sampling sites and visualised the results using boxplots. Mann–Whitney *U* tests were conducted at each sampling site to evaluate the statistical significance of differences between the DNase‐treated and untreated groups. Similarly, marine fish taxa, which are incapable of surviving in freshwater environments, were further analysed in parallel.

We identified specific taxa unique to either the DNase‐treated or untreated groups using Venn network diagrams. Subsequently, we focused on taxa recovered exclusively in untreated samples at each sampling site. We assessed their detection frequency across replicates (calculated as the number of detections in six parallel samples divided by the total number of detection attempts) and their relative abundance (defined as the proportion of sequences assigned to each species relative to the total sequences in a given sample). These characteristics can facilitate a conservative estimation of whether the detected taxa arose from random sampling variation or residual eDNA contamination. Taxa that were consistently detected across multiple replicates, exhibited relatively high abundance, or were ecologically implausible (e.g., marine or commercial species not native to the area) were considered likely to originate from residual eDNA contamination. In contrast, rare taxa detected sporadically and at very low abundance were more likely attributed to random sampling variation, although the possibility of residual eDNA contamination remains considerably high, particularly in the case of detected marine species.

A heat map was constructed based on the mean relative abundance of taxa per site, and hierarchical clustering was performed to explore how the omission of DNase treatment influenced taxon detection across different abundance thresholds (≥ 1%, 0.1%–1% and ≤ 0.1%; Wang et al. 2025a). Rarefaction curves were generated using resampled data to confirm that sequencing depth was sufficient across all 48 samples. All statistical analyses and visualisations were performed in R (version 4.4.2) (R Core Team 2023).

### 2.5 Community Composition Analysis

To evaluate the influence of residual eDNA on fish community composition, we employed a combination of multivariate statistical analyses. All analyses were performed in R (version 4.4.2; R Core Team 2023) using the vegan package. Firstly, non‐metric multidimensional scaling (NMDS) was used to visualise differences in fish community composition between DNase‐treated and untreated groups across the four sampling sites based on Bray–Curtis dissimilarity. Two‐dimensional NMDS plots were generated for each site, and stress values were reported to assess the reliability of the ordination results. To statistically evaluate the significance of community composition differences between DNase‐treated and untreated groups at each site, we conducted permutational multivariate analysis of variance (PERMANOVA) with 999 permutations using the *adonis2* function in the vegan package. Also, Similarity Percentage (SIMPER) analysis was performed using the *simper* function to calculate the degree of dissimilarity levels between DNase‐treated and untreated groups.

In the present study, the taxa with increased relative abundance in untreated samples were with distinct supplementary biological signals, which potentially originated from the residual eDNA in eRNA extracts, and the taxa with decreased relative abundance in untreated samples were with no or fewer supplementary biological signals. Thus, the taxa with increased relative abundance were screened and fold increase for these taxa/species in untreated groups were determined to explore the omission of DNase treatment influence on relative abundance of taxa. To avoid impacts of inherent changes of abundance in sequencing profiling in metabarcoding, the fold increase was considered to explore the variation in influences across four sampling sites. Also, the taxa additionally occurred in untreated samples were not considered to focus on abundance effects. The fold increase of taxa at each site was visualised by boxplots and corresponding Mann–Whitney *U* tests were performed with Bonferroni correction to assess whether the influence levels differed significantly across the sampling sites, especially at Site S3, which was located at the WWTP discharge point. Further, the fold increase of specific taxa was plotted using a heat map, allowing for a clear visualisation of variation across four sites.

## 3 Results

### 3.1 Overall Fish Biodiversity

From the DNase‐treated and untreated samples, a total of 46,870,132 raw reads were obtained. Following sequence assembly, 18,733,584 reads remained for quality control, resulting in 3,309,421 high‐quality reads. These were subsequently clustered into 141 ZOTUs. In total, 64 fish taxa were identified, including 5 to the family level, 13 to the genus level and 46 to the species level (Table S1). Rarefaction curve analysis confirmed that sequencing depth was sufficient to capture the taxonomic diversity within each sample (Figure S1).

### 3.2 Biodiversity Recovered From eRNA Metabarcoding With vs. Without DNase Treatment

**FIGURE 2:**
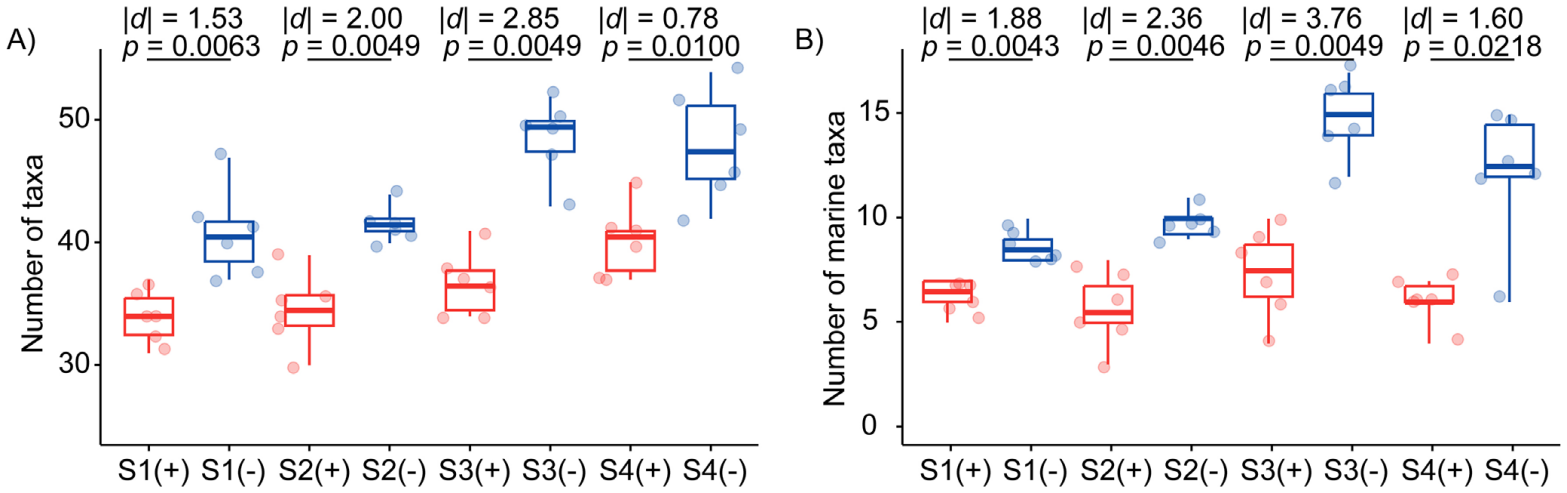
Comparison of (A) the total number of fish taxa and (B) the number of marine fish taxa detected from DNase‐treated (+) and untreated (−) eRNA samples across four sampling sites (S1–S4). Boxplots show variation among six biological replicates per treatment at each site. Statistical differences between treated and untreated groups were evaluated using Mann–Whitney *U* tests. |d|: The absolute effect size (Cliff's delta); *p*: The *p*‐value from the Mann–Whitney *U* test.

The comparison between DNase‐treated and untreated eRNA samples revealed a consistent and significant overestimation of fish biodiversity in the absence of DNase treatment. Across all four sampling sites, the number of detected taxa was significantly higher in the untreated samples (Figure 2A). Specifically, the average number of taxa recovered from DNase‐treated samples ranged from 34.0 to 40.2, while untreated samples recovered between 40.8 and 48.5 taxa. At each site, this increase in taxon richness was statistically significant (Figure 2A; *p* < 0.05, Mann–Whitney *U* test). The most pronounced differences were observed at Site S3, which is located at the WWTP discharge point, a known hotspot for eDNA pollution resulting from the release of treated effluent. An average of 48.5 taxa was recovered from the untreated samples, compared to only 36.7 in DNase‐treated samples, representing the largest discrepancy among all sites (Figure 2A; *p* < 0.01, Mann–Whitney *U* test). All these results clearly suggest the contribution of residual eDNA to species detection when the DNase treatment was omitted.

A parallel analysis focused on marine fish further supports the influence of eDNA residues. Across all sites, significantly more marine taxa were detected in untreated samples than in DNase‐treated ones (Figure 2B; *p* < 0.05, Mann–Whitney *U* test). For instance, at Site S3, we detected an average of 14.8 marine taxa (30.5% of the total detection taxa) from the untreated samples. Similarly, at Site S4 (downstream of the discharge point), 12.2 marine taxa (25.4% of the total detection taxa) were detected in the untreated samples. The consistent overrepresentation of marine species in untreated samples, particularly at and downstream of the WWTP discharge point, supports the presence and influence of residual eDNA originating from wastewater effluent.

**FIGURE 3:**
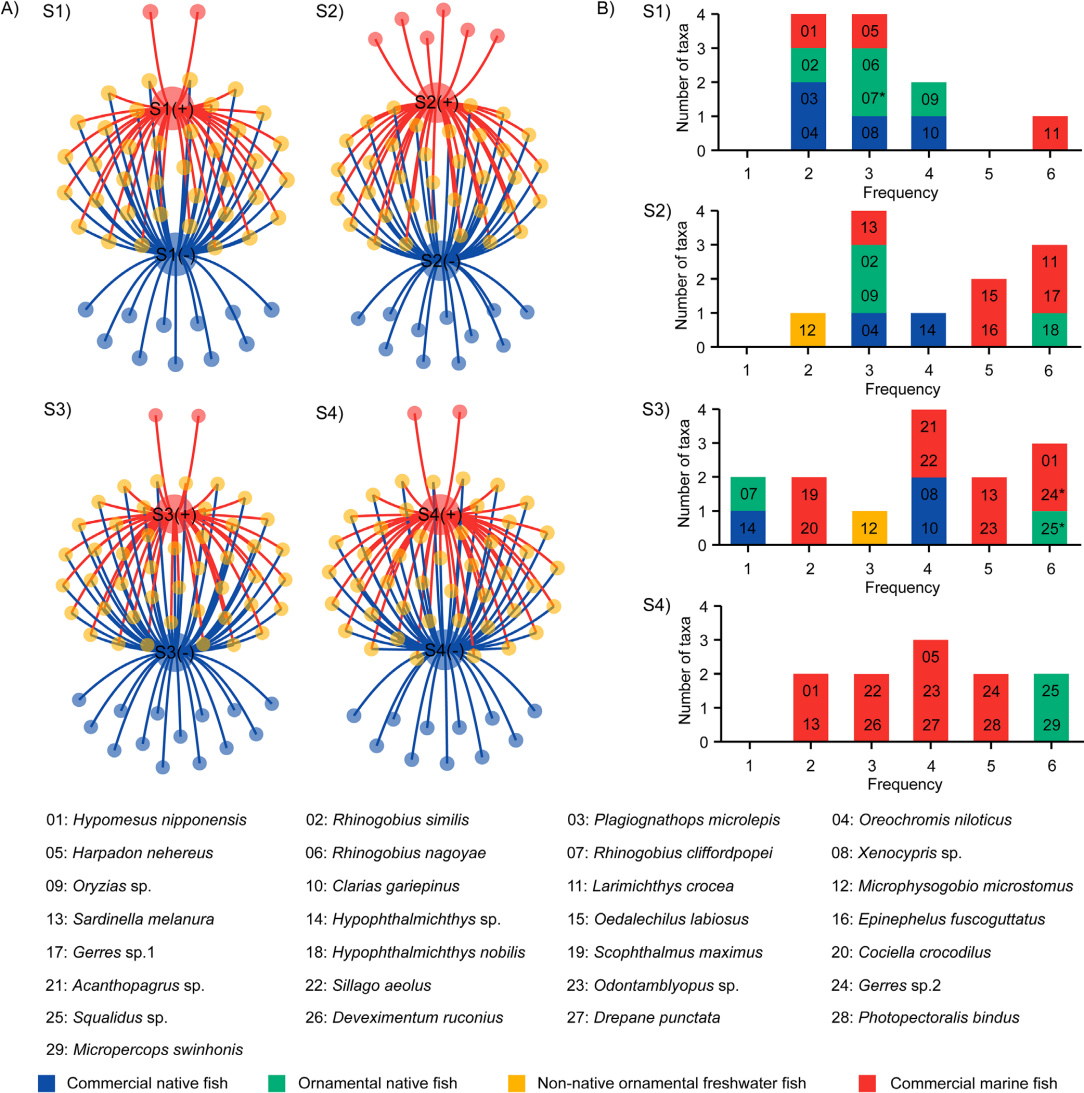
(A) Venn network diagram showing taxa occurrence in DNase‐treated and untreated groups across four sampling sites (S1–S4). Parent nodes (blue: untreated; red: treated) represent the two groups; yellow child nodes indicate shared species, blue nodes indicate taxa recovered only in the untreated group, and red nodes indicate taxa recovered only in the treated group. Edges connect taxa to their respective groups, with shared species linked to both parent nodes. (B) Taxonomic composition detected by eRNA metabarcoding without DNase treatment at Sites S1–S4 (* indicates taxa with a relative abundance > 0.1%).

Venn network diagram analysis revealed that the number of taxa specific to DNase‐treated samples was significantly lower than that specific to DNase‐untreated samples (Figure 3A). To further examine the influence of residual eDNA contamination, we analysed the specific fish taxa detected exclusively in DNase‐untreated samples across the four sampling sites. These taxa, which are absent in DNase‐treated counterparts, are strong candidates for being introduced by co‐extracted eDNA. Across all sites, a total of 29 additional taxa were uniquely detected in untreated samples (Figure 3B; Table S1). Of these, 16 were marine fish species, which are ecologically implausible in the freshwater river system, and 13 were freshwater taxa, among which 12 have been previously recorded in Beijing (Figure 3B). One freshwater species ( *Microphysogobio microstomus* ) was an ornamental fish never documented as native or non‐native in Beijing, suggesting potential contamination from aquarium‐related sources for wastewater (Figure 3B). When we examined these additional taxa at each sampling site, many taxa were detected at low relative abundances (< 0.1%) and low frequencies (≤ 2 out of 6 replicates), suggesting possible stochastic sampling effects. However, a notable proportion exhibited high detection frequency (≥ 3 replicates), and some had moderate to high relative abundance (> 0.1%), especially near or downstream of the wastewater treatment plant (WWTP). For example, 14 exclusive taxa were observed in the untreated group at Site S3 (WWTP discharge point), the highest among all sites. Six of the marine species and three freshwater species were detected in more than three replicates. Taxa, such as *Gerres* sp.2 and *Squalidus* sp., exhibited relative abundances > 0.1%. At Site S4 (downstream), 11 exclusive taxa were recovered from untreated samples, including nine marine and two freshwater species. Five of the marine taxa were detected in at least three replicates, suggesting non‐random occurrence, even though their relative abundances remained low (< 0.1%). In contrast, the two freshwater taxa were detected in all six replicates, indicating strong signals likely unrelated to random sampling effects (Figure 3B).

Given that Site S3, situated at the discharge point, represents an eDNA pollution hotspot, we anticipated detecting more pronounced eDNA signals, since trace amounts of eDNA at downstream sites may otherwise fall below the detection limit during eRNA metabarcoding. As expected, we detected five site‐specific taxa (Table S1): one native ornamental species (*Squalidus* sp.), which exhibited a relative abundance greater than 0.1% and was present in all six technical replicates, as well as four commercial marine fish species (*Odontamblyopus* sp., *Acanthopagrus* sp., *Sillago aeolus* and *Cociella crocodilus*), with detection frequencies of 5, 4, 4 and 2 replicates, respectively. The consistent detection and relatively high abundance of these taxa, especially the ecologically implausible marine species, strongly suggest that their presence in the untreated eRNA samples is attributable to residual eDNA contamination.

### 3.3 Influence of DNase Treatment on Taxa Detection Across Abundance Levels

**FIGURE 4:**
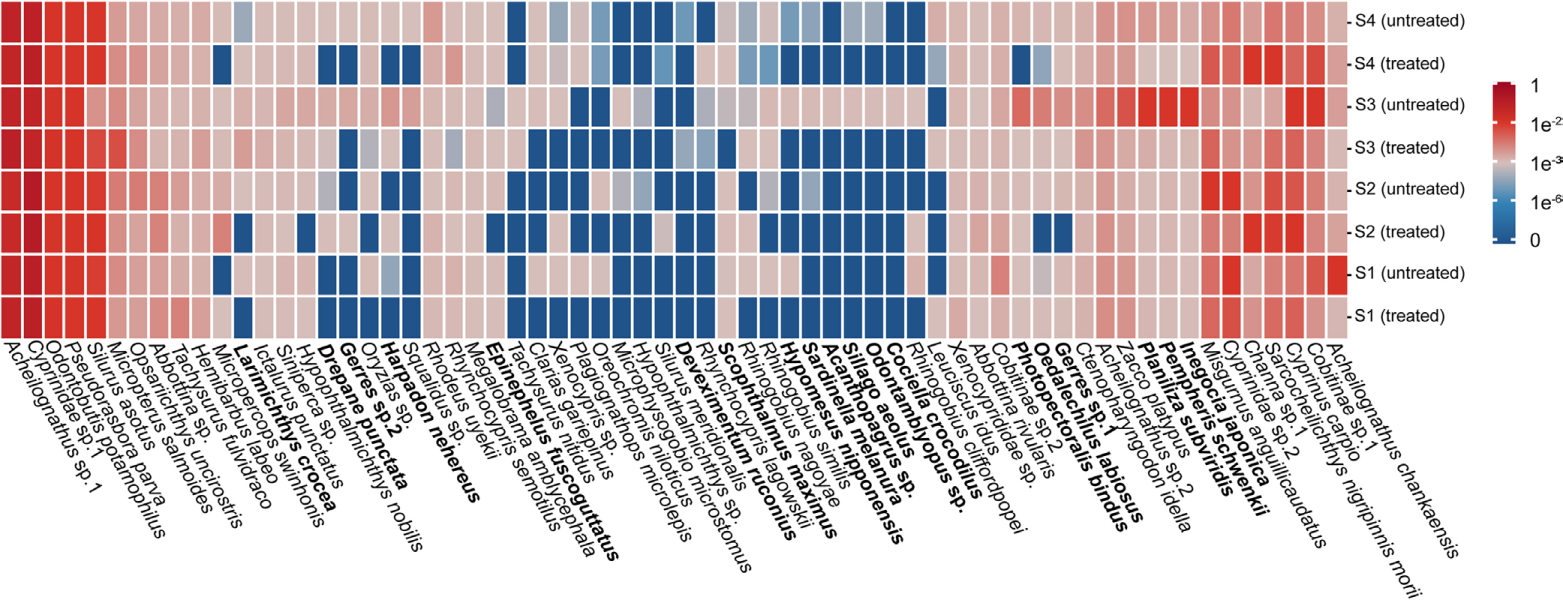
Comparison of fish community composition detected by eRNA metabarcoding with (+) and without (−) DNase treatment at Sites S1–S4. Species names of non‐native fish are shown in bold.

To better understand how DNase omission influenced taxa detection across abundance levels, we classified detected taxa into three categories: high (≥ 1%), moderate (0.1%–1%) and low (≤ 0.1%) relative abundance. At Site S1, no marine taxa were detected at moderate or high abundance levels in either the DNase‐treated or untreated groups. However, in the low‐abundance category, three additional marine species were detected exclusively in the untreated group (Figure 3B). The results at Site S2 were similar to those at Site S1, with four additional marine species and one non‐native species detected only in the untreated group at low abundance (Figure 3B). In contrast, at Sites S3 and S4, which were more strongly influenced by effluent discharge from WWTP, marine species were no longer confined to the low‐abundance category. At Site S3, three high‐abundance marine species, *Planiliza subviridis*, *Pempheris schwenkii* and *Inegocia japonica* , were detected in the untreated samples. These species were present at moderate, low and low abundance, respectively, in the DNase‐treated samples. Additionally, six moderate‐abundance marine taxa detected in the untreated samples were either reduced to low abundance or not detected at all in the DNase‐treated samples. Furthermore, seven low‐abundance marine species present in the untreated samples were completely undetected in the treated samples (Figure 4). At Site S4, three moderate‐abundance marine species were detected in the untreated group and the same three species observed at high abundance in the untreated samples at Site S3. In the DNase‐treated samples at Site S4, these three species were all reduced to low abundance. Moreover, nine low‐abundance marine species present in the untreated samples were completely undetected in the treated samples (Figure 4). The results demonstrate that omitting DNase treatment leads to the detection of false positives, even at moderate to high abundance levels, as supported by the marine taxa observed here.

### 3.4 Community Composition Variation Between Treated and Untreated Samples

**FIGURE 5:**
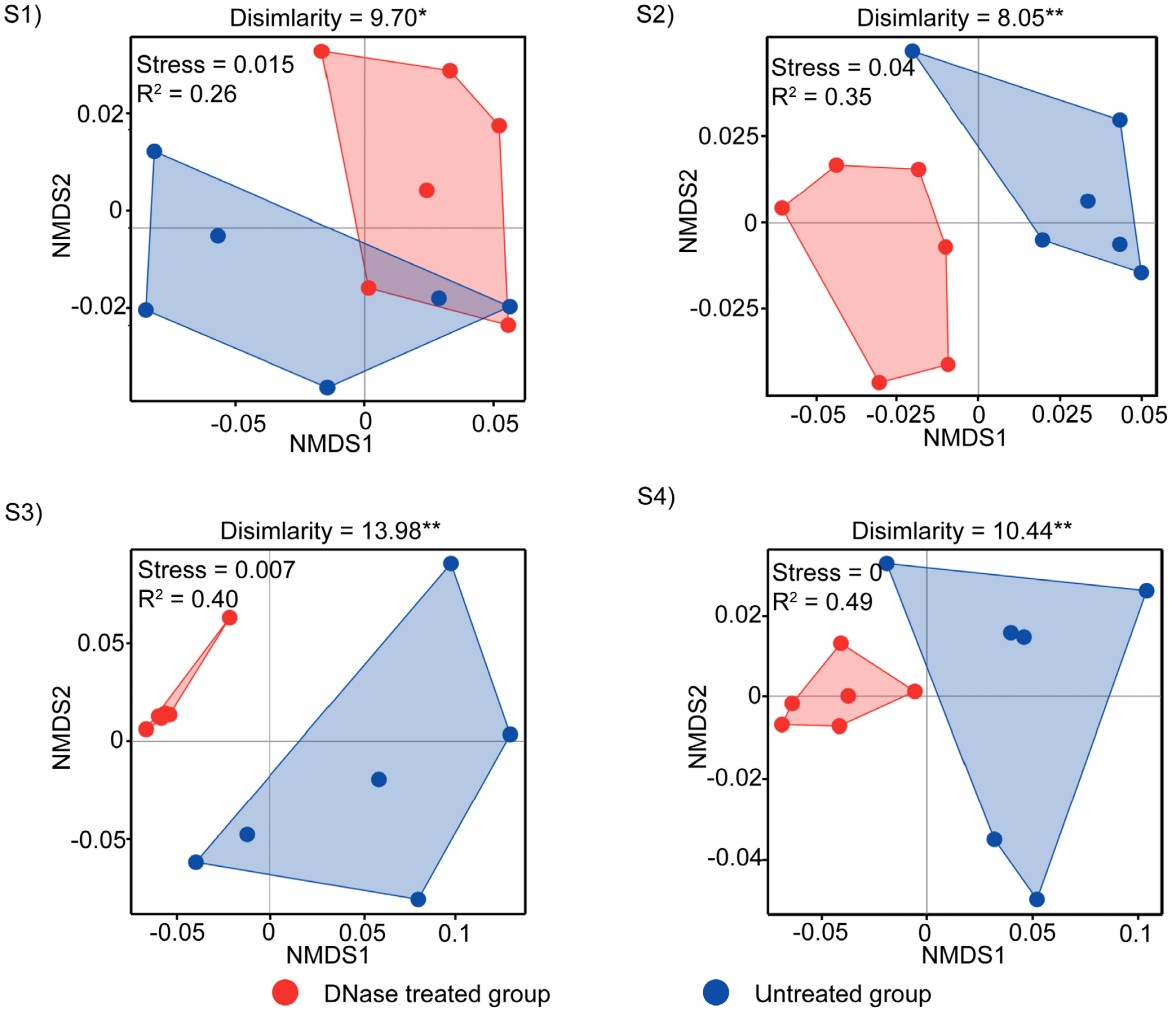
Differences in community composition between DNase‐treated and untreated groups across sampling sites (S1–S4). *p < 0.05 and **p < 0.01 based on permutational multivariate analysis of variance (PERMANOVA).

Community composition analyses revealed clear and consistent differences between DNase‐treated and untreated eRNA samples across all four sampling sites. Non‐metric multidimensional scaling (NMDS) ordinations showed that treated and untreated groups were distinctly clustered, with each group forming separate clusters at each site (Figure 5). These visual separations were statistically supported by PERMANOVA results, indicating significant differences in community composition between treated and untreated groups at all sites (S1–S4; *p* < 0.05). While the observed dissimilarity values between groups were moderate (S1: 9.70; S2: 8.05; S3: 13.98; S4: 10.44; Figure 5), they consistently reflected the influence of residual eDNA on taxonomic profiles in untreated samples. The main contributors to the dissimilarity at each site were listed in Table S2.

**FIGURE 6:**
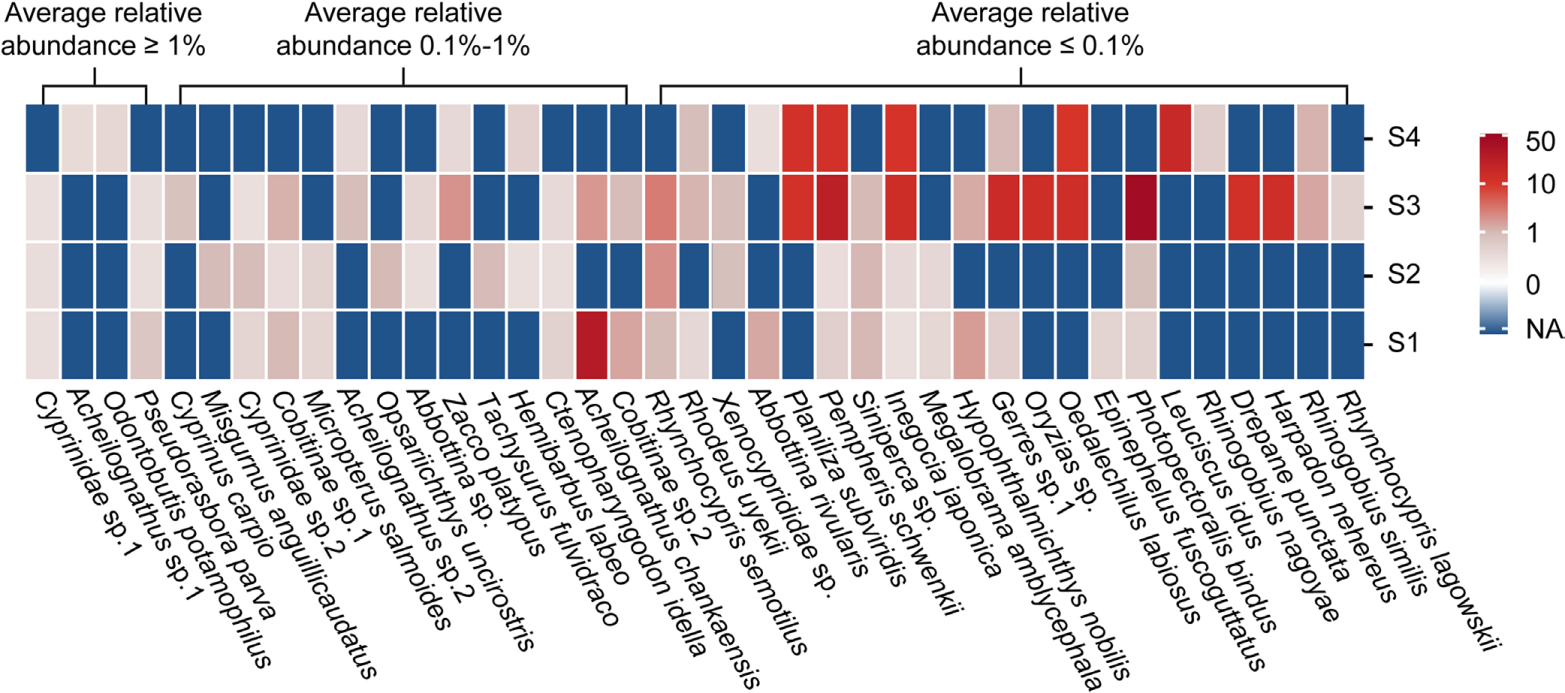
Fold increase of taxa with higher relative abundance in untreated groups across four sampling sites (S1–S4). Taxa are categorised by average abundance levels (≥ 1%, 0.1%–1% and ≤ 0.1%). ‘NA’ indicates taxa that either showed no increase in relative abundance or were not detected in either treatment group at a given site.

Given that changes in taxon abundance primarily accounted for the differences between DNase‐treated and untreated groups, we focused our analysis on taxa that showed elevated relative abundance in the untreated samples, as these are most likely influenced by residual eDNA. To minimise confounding effects arising from sequencing‐related variability in relative abundance estimates, we used fold‐change as the primary analytical metric, and raw data were normalised to percentage values prior to analysis. Consistent with the pronounced dissimilarity in taxonomic profiles between treated and untreated samples, Site S3 exhibited the highest overall fold increases among all sampling sites (Figure S2, *p* < 0.01), with nine taxa showing more than a 10‐fold increase. Site S4 (downstream) followed, with five taxa exhibiting similarly large increases. At Site S1, one taxon ( *Acheilognathus chankaensis* ) showed a striking 33‐fold increase, while all other taxa displayed more modest increases, with no greater than 3‐fold at S1 and 4‐fold at S2 (Figure 6). These results demonstrate that residual eDNA contamination can significantly distort eRNA‐derived abundance estimates, potentially leading to ecologically misleading interpretations of community structure.

## 4 Discussion

As eRNA metabarcoding gains traction in biodiversity monitoring, it becomes increasingly important to identify and address errors (false negatives and positives) that may compromise data accuracy (Littlefair et al. 2022; Zhan 2025). One of the overlooked concerns is the co‐extraction of eDNA during RNA isolation, a very common form of contamination that cannot be completely avoided despite the use of RNA‐specific extraction reagents and protocols. The residual DNA can be amplified during metabarcoding workflows following the reverse transcription of eRNA, leading to a high level of false positives in final biodiversity datasets. In this study, we clearly demonstrate that residual eDNA in eRNA extracts can substantially bias biodiversity estimates. By comparing DNase‐treated and untreated eRNA samples collected from a freshwater system, we found that the absence of DNase treatment led to inflated taxonomic richness (Figure 2A), spurious detection of non‐native (including marine) taxa (Figure 2B), and distorted community composition profiles (Figure 4). These results reveal a critical but often overlooked error source in eRNA metabarcoding workflows, thus emphasising the necessity of implementing effective DNase treatment protocols to ensure reliable interpretation of eRNA‐derived biodiversity data.

### 4.1 Common Presence of eDNA Residuals in Extracted eRNA

Indeed, numerous studies in medical, biological and evolutionary fields have demonstrated that even protocols specifically optimised for RNA purification fail to completely eliminate co‐extracted DNA (Chai et al. 2005; Nguyen et al. 2024). This is particularly true when working with low‐biomass or complex environmental samples, where extracellular DNA fragments are abundant and highly persistent in the surrounding matrix (Wang, Xiong, Liu, et al. 2025). While RNA‐specific extraction methods are designed to preferentially bind RNA and exclude genomic DNA, their ability to discriminate against small DNA fragments (e.g., those < 200 bp), which are common in degraded eDNA pools, is limited (Tavares et al. 2011; Li et al. 2022).

Indeed, evidence from transcriptomic studies highlights the extent of this DNA contamination issue. Comparative analyses of RNA and DNA co‐extraction workflows have shown that up to 20%–30% of reads in RNA‐derived libraries may originate from contaminating DNA (Stat et al. 2017; Marshall et al. 2021). These contamination levels are not trivial, particularly in eRNA metabarcoding, where false signals of biodiversity may arise not from actively transcribing organisms but from non‐living DNA sources that have persisted in the environment. The problem can be further magnified in aquatic or sedimentary environments, where DNA degrades more slowly than RNA due to protection within biofilms, adsorption to particles or reduced enzymatic activity (e.g., Turner et al. 2015). As a result, residual DNA in environmental samples is not only prevalent but also particularly resilient, making its co‐extraction during RNA isolation almost inevitable.

Our results provide clear evidence that residual eDNA is a common and impactful source of error in eRNA‐based biodiversity assessments. Across all sites, untreated eRNA samples detected significantly more taxa than DNase‐treated ones, with the largest inflation at the WWTP discharge point (Figure 2). Since eDNA concentrations can be elevated at the effluent discharge point, the greater number of false positive taxa observed there suggests that sites with higher eDNA concentrations are more prone to false positives caused by residual eDNA. Many additional taxa were ecologically implausible marine or ornamental species, detected at moderate abundance and in multiple replicates, strongly indicating contamination from co‐extracted eDNA. These findings confirm that even with standard protocols, small DNA fragments can persist and distort biodiversity metrics, emphasising the need for rigorous treatment and contamination control in all eRNA workflows.

### 4.2 Technical Challenge in Distinguishing Taxa Derived From eDNA Residuals

Due to the extreme sensitivity of metabarcoding, even trace amounts of residual eDNA can be co‐amplified alongside cDNA generated from reverse transcribed eRNA, resulting in false biodiversity signals. A major challenge in eRNA metabarcoding is reliably distinguishing true biological signals derived from eRNA transcripts from contamination caused by residual eDNA in environmental samples. Because eDNA and transcribed cDNA are highly similar in nature, discriminative approaches, such as using biological or technical replicates (Zhan, He, et al. 2014; Xiong et al. 2016) or employing RT‐minus controls (i.e., reverse transcription without reverse transcriptase; Del Aguila et al. 2005), are often insufficient to reliably and definitively distinguish taxa derived from residual eDNA contamination. For the strategy of biological or technical replicates, RNA‐specific extraction protocols often lead to low abundance of residual eDNA. The common occurrence of stochasticity (i.e., random sampling errors) that causes inconsistent detection of rare taxa (Zhan et al. 2013; Zhan, Xiong, et al. 2014) makes it impossible to distinguish taxa originating from residual eDNA versus those genuinely derived from rare eRNA transcripts using replicates alone. Indeed, our results demonstrate that residual eDNA affected not only low‐abundance taxa but also taxa with moderate and high abundance (Figure 4). While RT‐minus controls can indicate the presence of residual DNA (e.g., Del Aguila et al. 2005), they are insufficient for reliably attributing taxonomic signals to either active eRNA transcripts or contaminating eDNA. This arises because taxa detected in RT‐minus controls confirm residual DNA presence, but the absence of a taxon in these controls does not guarantee that its corresponding signal in RT‐positive samples originates exclusively from RNA. This is especially true when DNA and RNA signals overlap or when DNA is present below the detection threshold. Moreover, trace amounts of residual eDNA often persist in eRNA extracts from environmental samples, even after DNase treatment (see below 4.4 for more details). Since the downstream PCR step amplifies both residual eDNA and cDNA generated from RNA, distinguishing their respective contributions remains impossible.

In this study, we addressed this technical challenge using a field‐based design focused on a freshwater river system receiving treated effluent from a major WWTP (Figure 1). While we did not directly sequence the effluent, previous research has demonstrated that treated wastewater is largely devoid of eRNA while potentially harbouring high loads of legacy eDNA (Xiong et al. 2024). This is supported by the nature of wastewater treatment, where advanced oxidation and microbial digestion are highly effective at degrading labile RNA molecules (Xiong et al. 2024). Furthermore, our results demonstrate that marine taxa, likely originating from human dietary sources in wastewater, were detected almost exclusively in samples that did not undergo DNase treatment. The near‐absence of these taxa in DNase‐treated eRNA libraries suggests their detection resulted from eDNA carryover rather than intact eRNA. Consequently, this system provides a rigorous ‘worst‐case’ environment to evaluate how residual eDNA can distort biodiversity metrics if not properly removed.

### 4.3 Impacts of Residual eDNA on eRNA‐Based Studies

The presence of residual eDNA in eRNA extracts represents a critical methodological challenge that can substantially compromise the accuracy of biodiversity assessments. Our findings demonstrate that untreated eRNA samples consistently detected significantly more taxa than DNase‐treated counterparts (Figure 2). The impacts of inflation of species richness are obvious. As eRNA‐based biodiversity assessments are often used to illustrate recent biological activities, such inflation can lead to misinterpretation of current ecosystem dynamics by falsely indicating the presence of taxa that are no longer active or present. This compromises the ability to accurately monitor temporal changes, assess ecosystem health and make informed management decisions.

The impacts of residual eDNA are particularly problematic for rare or low‐abundance taxa, where the boundary between authentic biological signals and contamination becomes blurred. Our detection of ornamental fish species at relative abundances < 0.1% in untreated samples highlights this challenge, echoing concerns raised in eDNA studies about distinguishing rare species from contamination (Zhan, Xiong, et al. 2014; Stat et al. 2017). This issue becomes even more pronounced in complex environmental matrices or highly biodiverse systems, where the sheer volume of background DNA increases the likelihood of false positives, as demonstrated at Site S3 in this study (Figure 3). Such challenges are particularly critical in applications, such as evaluating the eradication of invasive species. False detection of an invasive species as actively present due to residual eDNA contamination could trigger further unnecessary management actions or divert resources away from actual ecological threats, potentially resulting in inefficient allocation of conservation efforts and undermining the credibility of monitoring programs (Lodge et al. 2012; Ficetola et al. 2015).

Beyond simple taxon counts, residual eDNA can significantly distort community composition metrics, as observed in our study where untreated samples showed more than 10‐fold abundance increases for certain species (Figure 6). Inflated read abundances can cause certain taxa to appear disproportionately dominant, while masking the presence of genuinely rare or low‐abundance species. Such biases are particularly problematic in systems where abundance‐based information is used to infer ecological condition, monitor anthropogenic impacts or guide conservation priorities. For instance, bioassessment frameworks that rely on the relative abundance of pollution‐tolerant or indicator taxa may misclassify ecosystem health if their signal is artificially elevated due to residual eDNA. The same risk applies to studies investigating environment–community interactions, where the relative abundance of taxa is often used to infer ecological responses to environmental gradients such as nutrient loading or pollution. In such cases, inflated abundance estimates caused by residual DNA can weaken or distort observed correlations, leading to incorrect conclusions about ecological drivers or stressor impacts. For a statistical aspect, multivariate analyses commonly used in ecological studies, including NMDS or PERMANOVA, are also sensitive to outliers and abundance‐weighted variation; thus, eRNA without DNase treatment may obscure real spatial or temporal patterns by inflating similarity among distinct communities or introducing artificial gradients.

### 4.4 Optimising DNase Treatment for eRNA Workflows

Even with RNA‐specific extraction protocols, fragmented eDNA can escape from standard binding and washing steps, leading to its unintended carryover into eRNA extracts. This underscores the inherent challenge of fully eliminating eDNA contamination during the RNA extraction step. Consequently, best practices for eRNA workflows emphasise a post‐extraction, strict contamination control strategy. DNase treatment, when optimised in terms of enzyme concentration, incubation time and reaction conditions, effectively reduces the DNA load without compromising RNA integrity. Multiple studies across diverse sample types, including bacterial cultures, whole blood and plant tissues, have demonstrated that optimised DNase I treatment can effectively remove contaminating DNA from RNA extracts. For example, incubating 1 μg of RNA with 1 U of DNase I at 37°C for 30 min, followed by heat inactivation at 75°C for 5 min, efficiently eliminated residual DNA while preserving mRNA quality (Huang et al. 1996). However, a single DNase I digestion often failed to remove all residual genomic DNA detectable by highly sensitive qPCR. Implementing a second DNase digestion combined with thorough purification (e.g., via optimised workflows prior to enzymatic steps) reliably reduces DNA contamination to below detectable levels without significantly compromising RNA integrity (Lim et al. 2016; Páska et al. 2019). Similarly, although we applied an excess of DNase in our protocol, we still detected marine species from the river ecosystem (Figure 2B and Table S1). This residual detection likely stems from multiple factors: incomplete removal of eDNA by a single‐step DNase treatment and/or the presence of genuine eRNA sources, such as untreated effluent or residual tissues from commercial fish species. However, contamination during laboratory procedures is unlikely, as blank controls were included at every step, and no valid fish taxa were detected in any of these controls. Therefore, further technical evaluation of DNase treatment parameters, including enzyme concentration, incubation time and the number of digestion cycles, is essential to ensure thorough removal of residual eDNA. However, overly aggressive DNase treatment may compromise eRNA integrity (Páska et al. 2019), highlighting the need to carefully balance DNA removal efficiency with RNA preservation. To enhance the reliability of DNA removal workflows, we recommend the integration of complementary validation tools. Known RNA spike‐ins should be employed as internal standards to quantify recovery rates and monitor for potential RNA degradation or enzymatic inhibition during the DNase and reverse transcription stages. Simultaneously, mock communities comprising known ratios of DNA and RNA from non‐target taxa can be utilised as benchmarks to directly measure the efficiency of DNase digestion. While spike‐ins monitor the integrity of the existing eRNA throughout the DNase treatment and reverse transcription stages, mock communities provide a quantitative benchmark to directly measure the efficiency of DNA removal.

While our study demonstrates the critical impact of residual eDNA on eRNA‐based biodiversity estimates and underscores the necessity of DNase treatment, it is important to note that only a single DNase treatment condition was evaluated here. We did not perform a systematic comparison of alternative DNA removal methods or commercially available kits, such as the PrimeScript RT Reagent Kit with gDNA Eraser (Jo 2023). Therefore, although our results strongly support the inclusion of DNA removal steps in eRNA workflows, the optimal protocol, including enzyme type, concentration, incubation time and number of digestion cycles, remains to be fully established. Future research should systematically compare multiple DNase treatments and other DNA‐removal strategies across diverse environmental matrices (e.g., freshwater, soils, sediments and air samples) to develop standardised workflows that maximise DNA removal efficiency without compromising RNA integrity.

## 5 Conclusions

Our study provides clear evidence that residual eDNA in eRNA extracts can cause substantial overestimation of species richness and significant distortion of community composition in eRNA‐based biodiversity assessments. The detection of ecologically implausible taxa, such as marine fish in a freshwater river, highlights the severity of false positives introduced when DNase treatment is omitted. False positives from eDNA residues were most pronounced at sites with high eDNA concentrations, such as the wastewater discharge point, suggesting that elevated eDNA concentrations increase the risk of false positive detections due to residual DNA. Based on these findings, we recommend that eRNA workflows always include DNase treatment, with its efficiency verified using sensitive qPCR assays. Where necessary, duplicate or multiple digestion cycles should be considered to ensure thorough DNA removal while preserving RNA integrity.

While our study utilised an aquatic model, the necessity of rigorous DNase treatment extends to other environmental matrices, such as soils, sediments and air, each presenting distinct challenges. In soil and sediment samples, the presence of humic substances and high mineral content can significantly inhibit DNase activity or physically sequester eDNA within aggregates, potentially requiring increased enzyme concentrations or surfactants to ensure complete digestion. Similarly, air samples often contain low‐concentration eRNA susceptible to rapid degradation, where the balance between effective DNA removal and maintaining RNA integrity is even more delicate. Thus, we emphasise that researchers should validate DNase efficiency specifically for their target matrix, as inhibitory compounds inherent to these complex environments can lead to incomplete DNA removal and subsequent biodiversity bias.

## Author Contributions

Conceptualization, A.Z.; methodology, F.W., W.X., X.H., A.Z.; formal analyses, F.W., W.X., X.H.; investigation, W.X., X.H., S.L., A.Z.; resources, W.X., X.H., S.L., A.Z.; writing – original draft, all co‐authors; writing – review and editing, all coauthors; project administration, A.Z.; funding acquisition, W.X., X.H., S.L., A.Z.

## Funding

This work was supported by Jing‐Jin‐Ji Regional Integrated Environmental Improvement—National Science and Technology Major Project (2025ZD1207600, 2025ZD1200800), the National Key R&D Program of China (No. 2021YFC3200102), Guiding Funds of Central Government for Supporting the Development of Local Science and Technology (2024ZY0128), National Natural Science Foundation of China (32471608).

## Conflicts of Interest

The authors declare no conflicts of interest.

## Supporting information


**Figure S1:** Rarefaction curves of detected fish taxa per sample using eRNA metabarcoding with and without DNase treatment across four sampling sites. Each treatment at each site includes six replicates.
**Figure S2:** Fold increase in relative abundance of taxa affected by the omission of DNase treatment across sampling sites (S1–S4). Statistical significance of variation was assessed using *p*‐value (Mann–Whitney *U* test).
**Table S1:** Fish taxa detected by eRNA metabarcoding with (+) and without (−) DNase treatment across four sampling sites (S1–S4). ‘Y’ denotes detection, and ‘N’ denotes non‐detection. Taxa are colour‐coded by relative abundance: red for high (> 1%), orange for moderate (0.1%–1%) and green for low (< 0.1%).
**Table S2:** Similarity percentage analysis (SIMPER, average dissimilarity, contribution) for detected by eRNA metabarcoding with (+) and without (−) DNase treatment across four sampling sites (S1–S4).

## Data Availability

The data that support the findings of this study are openly available in NCBI GenBank at https://www.ncbi.nlm.nih.gov/, reference number SRP650939.
