# Supplementary material for: Residual eDNA in eRNA Extracts Skews eRNA‐Based Biodiversity Assessment: Call for Optimised DNase Treatment
Source: Mol Ecol Resour. 2026 Jan 19;26(2):e70102. doi: 10.1111/1755-0998.70102 (PMC12813962; doi:10.1111/1755-0998.70102)
Supplement: Supplementary file 1 — Figure S1: Rarefaction curves of detected fish taxa per sample using eRNA metabarcoding with and without DNase treatment across four sampling sites. Each treatment at each site includes six replicates. Figure S2: Fold increase in relative abundance of taxa affected by the omission of DNase treatment across sampling sites (S1–S4). Statistical significance of variation was assessed using p‐value (Mann–Whitney U test). Table S1: Fish taxa detected by eRNA metabarcoding with (+) and without (−) DNase treatment across four sampling sites (S1–S4). ‘Y’ denotes detection, and ‘N’ denotes non‐detection. Taxa are colour‐coded by relative abundance: red for high (> 1%), orange for moderate (0.1%–1%) and green for low (< 0.1%). Table S2: Similarity percentage analysis (SIMPER, average dissimilarity, contribution) for detected by eRNA metabarcoding with (+) and without (−) DNase treatment across four sampling sites (S1–S4). [file MEN-26-e70102-s001.docx]

**Supplemental Table S1** Fish taxa detected by eRNA metabarcoding with (+) and without (−) DNase treatment across four sampling sites (S1-S4). “Y” denotes detection, and “N” denotes non-detection. Taxa are color-coded by relative abundance: red for high (>1%), orange for moderate (0.1%-1%), and green for low (<0.1%).

| species | S1(+) | S1(-) | S2(+) | S2(-) | S3(+) | S3(-) | S4(+) | S4(-) |
| --- | --- | --- | --- | --- | --- | --- | --- | --- |
| *Abbottina rivularis* | Y | Y | Y | Y | Y | Y | Y | Y |
| *Abbottina* sp. | Y | Y | Y | Y | Y | Y | Y | Y |
| *Acanthopagrus* sp. | N | N | N | N | N | Y | N | N |
| *Acheilognathus chankaensis* | Y | Y | Y | Y | Y | Y | Y | Y |
| *Acheilognathus* sp.1 | Y | Y | Y | Y | Y | Y | Y | Y |
| *Acheilognathus* sp.2 | Y | Y | Y | Y | Y | Y | Y | Y |
| *Channa* sp.1 | Y | Y | Y | Y | Y | Y | Y | Y |
| *Clarias gariepinus* | N | Y | N | N | N | Y | Y | Y |
| *Cobitinae* sp.1 | Y | Y | Y | Y | Y | Y | Y | Y |
| *Cobitinae* sp.2 | Y | Y | Y | Y | Y | Y | Y | Y |
| *Cociella crocodilus* | N | N | N | N | N | Y | N | N |
| *Ctenopharyngodon idella* | Y | Y | Y | Y | Y | Y | Y | Y |
| *Cyprinidae* sp.1 | Y | Y | Y | Y | Y | Y | Y | Y |
| *Cyprinidae* sp.2 | Y | Y | Y | Y | Y | Y | Y | Y |
| *Cyprinus carpio* | Y | Y | Y | Y | Y | Y | Y | Y |
| *Deveximentum ruconius* | N | N | N | N | Y | N | N | Y |
| *Drepane punctata* | N | N | Y | Y | Y | Y | N | Y |
| *Epinephelus fuscoguttatus* | Y | Y | N | Y | Y | Y | Y | Y |
| *Gerres* sp.1 | Y | Y | N | Y | Y | Y | Y | Y |
| *Gerres* sp.2 | N | N | Y | N | N | Y | N | Y |
| *Harpadon nehereus* | N | Y | Y | N | Y | Y | N | Y |
| *Hemibarbus labeo* | Y | Y | Y | Y | Y | Y | Y | Y |
| *Hypomesus nipponensis* | N | Y | N | N | N | Y | N | Y |
| *Hypophthalmichthys nobilis* | Y | Y | N | Y | Y | Y | Y | Y |
| *Hypophthalmichthys* sp. | N | N | N | Y | N | Y | N | N |
| *Ictalurus punctatus* | Y | Y | Y | Y | Y | Y | Y | Y |
| *Inegocia japonica* | Y | Y | Y | Y | Y | Y | Y | Y |
| *Larimichthys crocea* | N | Y | N | Y | Y | Y | Y | Y |
| *Leuciscus idus* | Y | N | N | N | Y | N | Y | Y |
| *Megalobrama amblycephala* | Y | Y | Y | Y | Y | Y | Y | Y |
| *Micropercops swinhonis* | Y | N | Y | Y | Y | Y | N | Y |
| *Microphysogobio microstomus* | N | N | N | Y | N | Y | N | N |
| *Micropterus salmoides* | Y | Y | Y | Y | Y | Y | Y | Y |
| *Misgurnus anguillicaudatus* | Y | Y | Y | Y | Y | Y | Y | Y |
| *Odontamblyopus* sp. | N | N | N | N | N | Y | N | Y |
| *Odontobutis potamophilus* | Y | Y | Y | Y | Y | Y | Y | Y |
| *Oedalechilus labiosus* | Y | Y | N | Y | Y | Y | Y | Y |
| *Opsariichthys uncirostris* | Y | Y | Y | Y | Y | Y | Y | Y |
| *Oreochromis niloticus* | N | Y | N | Y | N | N | Y | Y |
| *Oryzias* sp. | N | Y | N | Y | Y | Y | Y | Y |
| *Pempheris schwenkii* | Y | Y | Y | Y | Y | Y | Y | Y |
| *Photopectoralis bindus* | Y | Y | Y | Y | Y | Y | N | Y |
| *Plagiognathops microlepis* | N | Y | N | N | N | N | Y | Y |
| *Planiliza subviridis* | Y | Y | Y | Y | Y | Y | Y | Y |
| *Pseudorasbora parva* | Y | Y | Y | Y | Y | Y | Y | Y |
| *Rhinogobius cliffordpopei* | N | Y | Y | N | N | Y | N | N |
| *Rhinogobius nagoyae* | N | Y | Y | N | Y | Y | Y | Y |
| *Rhinogobius similis* | N | Y | N | Y | Y | Y | Y | Y |
| *Rhodeus uyekii* | Y | Y | Y | Y | Y | Y | Y | Y |
| *Rhynchocypris lagowskii* | N | N | N | N | Y | Y | Y | N |
| *Rhynchocypris semotilus* | Y | Y | Y | Y | Y | Y | Y | Y |
| *Sarcocheilichthys nigripinnis morii* | Y | Y | Y | Y | Y | Y | Y | Y |
| *Sardinella melanura* | N | N | N | Y | N | Y | N | Y |
| *Scophthalmus maximus* | Y | Y | Y | Y | N | Y | Y | Y |
| *Sillago aeolus* | N | N | N | N | N | Y | N | Y |
| *Silurus asotus* | Y | Y | Y | Y | Y | Y | Y | Y |
| *Silurus meridionalis* | N | N | Y | N | N | N | Y | N |
| *Siniperca* sp. | Y | Y | Y | Y | Y | Y | Y | Y |
| *Squalidus* sp. | N | N | N | N | N | Y | N | Y |
| *Tachysurus fulvidraco* | Y | Y | Y | Y | Y | Y | Y | Y |
| *Tachysurus nitidus* | N | N | N | N | Y | Y | N | N |
| *Xenocyprididae* sp. | Y | Y | Y | Y | Y | Y | Y | Y |
| *Xenocypris* sp. | N | Y | Y | N | N | Y | Y | Y |
| *Zacco platypus* | Y | Y | Y | Y | Y | Y | Y | Y |

**Supplemental Table S2** Similarity percentage analysis (SIMPER, average dissimilarity, contribution) for detected by eRNA metabarcoding with (+) and without (−) DNase treatment across four sampling sites (S1-S4).

| site | taxa | contribution% |
| --- | --- | --- |
| S1 (DNA vs. RNA) dissimilarity = 9.70 | *Acheilognathus* sp.1 | 22.71 |
|  | *Cyprinidae* sp.1 | 16.63 |
|  | *Acheilognathus chankaensis* | 12.60 |
|  | *Pseudorasbora parva* | 8.54 |
|  | *Odontobutis potamophilus* | 8.35 |
| S2 (DNA vs. RNA) dissimilarity = 8.05 | *Cyprinidae* sp.1 | 26.00 |
|  | *Acheilognathus* sp.1 | 16.56 |
|  | *Silurus asotus* | 9.20 |
|  | *Channa sp.1* | 6.55 |
|  | *Odontobutis potamophilus* | 6.29 |
| S3 (DNA vs. RNA) dissimilarity = 13.98 | *Acheilognathus* sp.1 | 27.31 |
|  | *Cyprinidae* sp.1 | 12.94 |
|  | *Pempheris schwenkii* | 6.76 |
|  | *Odontobutis potamophilus* | 6.28 |
|  | *Planiliza subviridis* | 6.24 |
| S4 (DNA vs. RNA) dissimilarity = 10.44 | *Acheilognathus* sp.1 | 32.93 |
|  | *Cyprinidae* sp.1 | 28.21 |
|  | *Silurus asotus* | 4.86 |
|  | *Pseudorasbora parva* | 4.25 |
|  | *Channa* sp.1 | 3.83 |


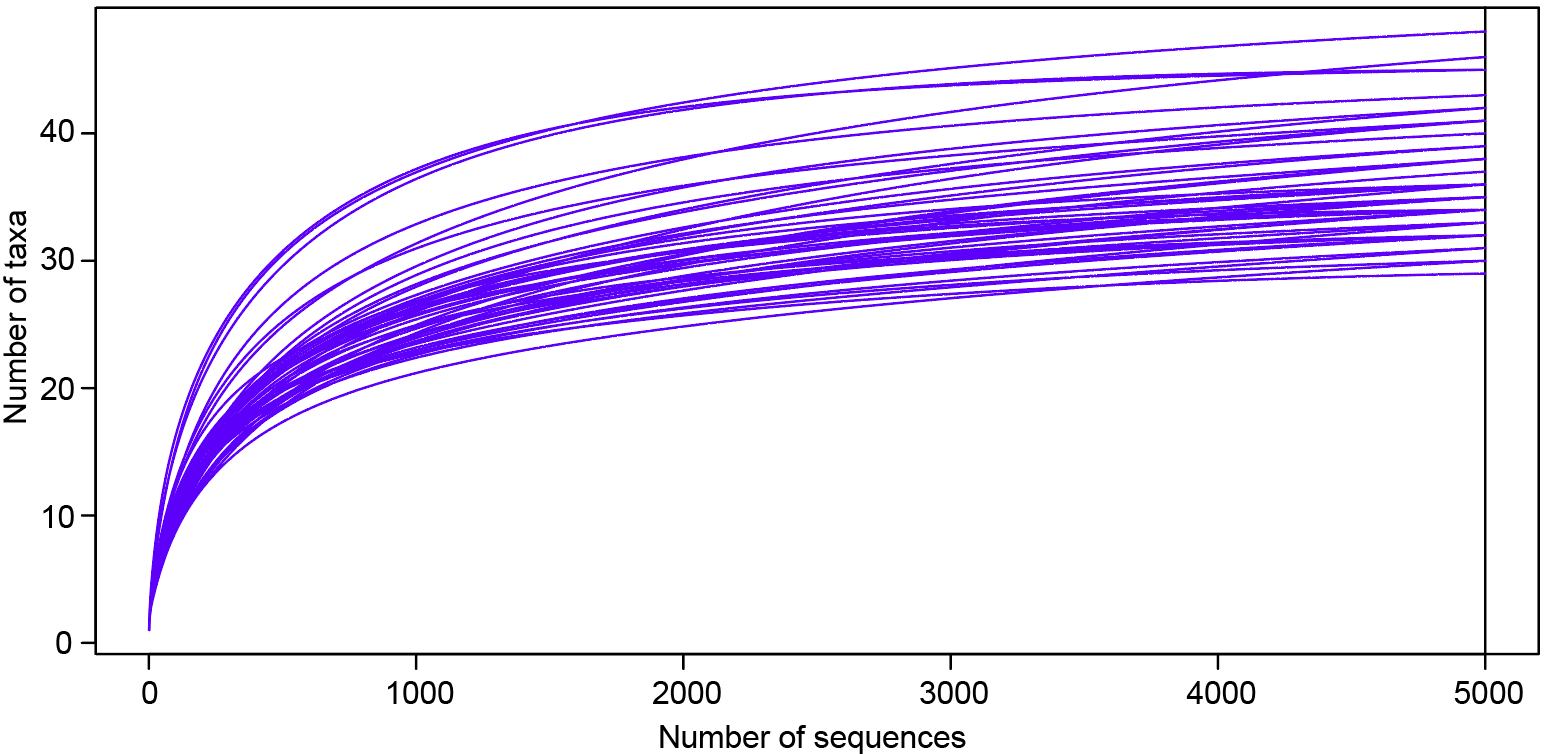


**Figure S1** Rarefaction curves of detected fish taxa per sample using eRNA metabarcoding with and without DNase treatment across four sampling sites. Each treatment at each site includes six replicates.


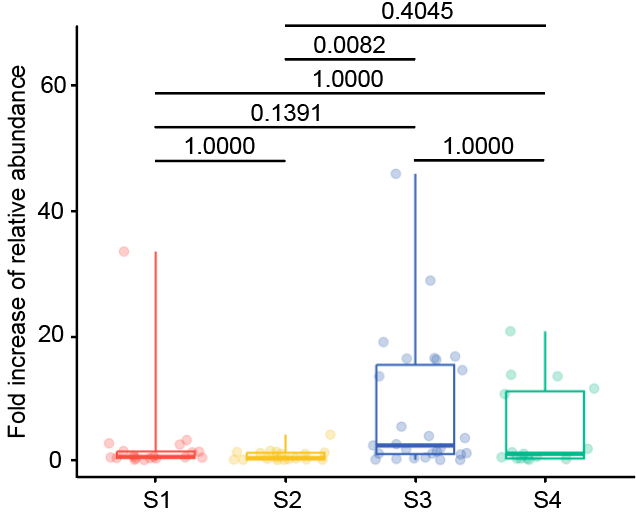


**Figure S2** Fold increase in relative abundance of taxa affected by the omission of DNase treatment across sampling sites (S1-S4). Statistical significance of variation was assessed using *p*-value (Mann-Whitney *U* test).
